# Supplementary material for: Genetic comparisons of interleukin-17 reveal a framework for complex signaling evolution
Source: bioRxiv. 2026 Apr 14:2026.04.13.718218. Preprint. [Version 1] doi: 10.64898/2026.04.13.718218 (PMC13105070; doi:10.64898/2026.04.13.718218)
Supplement: Supplement 3 [file media-3.zip › Supplemental files for paper/ERC/IL17xIL17_comparisons/IL17RC_IL17RC.pdf]

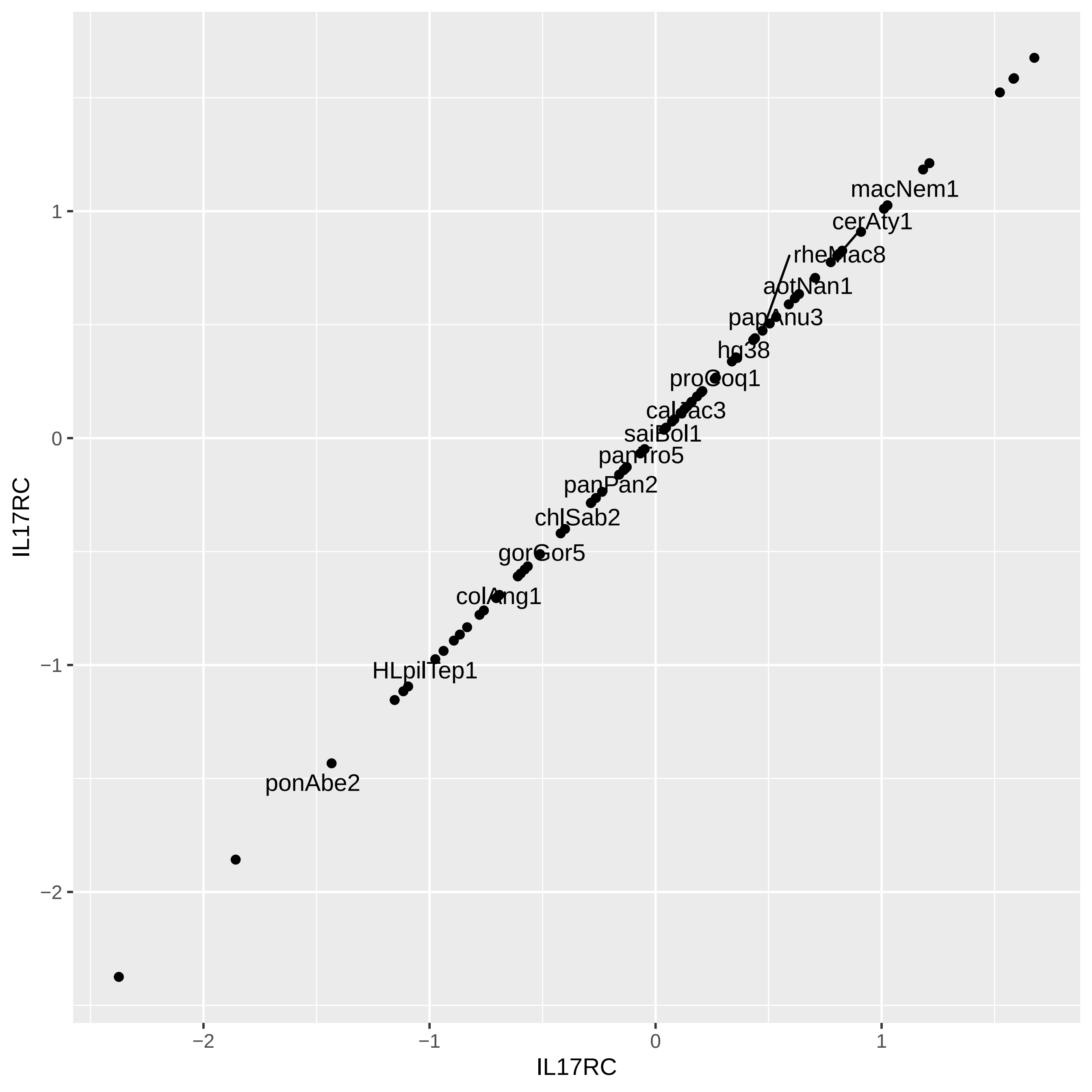

macNem1
cerAty1
rheMac8
aotNan1
papAnu3
hg38
proCoq1
calJac3
saiBol1
panTro5
panPan2
chlSab2
gorGor5
colAng1
HLpilTep1
ponAbe2
1
0
−1
−2
IL17RC
IL17RC
